# Endovascular management of a large renal artery aneurysm: a case report and review of the literature

**DOI:** 10.1186/s12894-021-00877-6

**Published:** 2021-09-07

**Authors:** Alec Zhu, Peter Connolly, A. Ari Hakimi

**Affiliations:** 1 NewYork-Presbyterian/Weill Cornell Medical Center, 525 E 68th St, New York, NY 10065 USA; 2 Memorial Sloan Kettering Cancer Center, 1275 York Ave, New York, NY 10065 USA

**Keywords:** Renal, Artery, Aneurysm, Pseudoaneurysm, Case report

## Abstract

**Background:**

A renal artery aneurysm is a rare clinical presentation that can be found incidentally on imaging or during workup for refractory hypertension. Its presentation can be similar to that of a renal artery pseudoaneurysm, but the etiologies of the two vascular lesions differ. We present a patient who had an incidental finding of a large renal artery aneurysm that was managed with endovascular embolization. We also describe the literature surrounding the etiology, presentation and management of both renal artery aneurysms and renal artery pseudoaneurysms.

**Case presentation:**

A 62-year-old man was referred to a urologic oncologist for workup of a newly found renal mass. Initial imaging with computed tomography showed a homogenous, well-circumscribed mass arising from the right kidney. Further evaluation with Doppler ultrasonography demonstrated pulsatile flow within the renal mass that was concerning for a renal artery pseudoaneurysm. The patient initially underwent a diagnostic angiogram by interventional radiology and was found to have a true renal artery aneurysm. Interventional radiology considered placement of a covered stent or angioembolization, but treatment was deferred due to concern for compromising the patient’s renal function. Patient was subsequently transferred to a neighboring hospital for management by vascular surgery. After considering both open surgical and endovascular approaches, the patient ultimately underwent angioembolization of the renal artery aneurysm. Short-term follow-up showed successful exclusion of the aneurysm with minimal adverse effects to the patient.

**Conclusions:**

Our case report documents a unique case of an incidentally found large renal artery aneurysm that was successfully managed with endovascular embolization. Renal artery aneurysms and renal artery pseudoaneurysms, which can present similarly on imaging, are important diagnostic considerations in a patient presenting with a new renal mass. While open surgical approaches can be used to repair aneurysms, endovascular approaches using stenting or angioembolization are safe and effective options for treating renal aneurysms and renal pseudoaneurysms.

## Background

A renal artery aneurysm (RAA) is a rare vascular malformation that is typically found incidentally on imaging [1]. On rare occasions, RAAs can rupture and cause a life-threatening hemorrhage with a mortality rate approaching 10%, and this risk is significantly higher in pregnant women [2, 3]. The RAA is characterized by a localized defect in the internal elastic tissue that allows it to dilate; a true aneurysm contains all three layers of the arterial wall [4]. In contrast, a renal artery pseudoaneurysm (RAP) is a hematoma that forms from an arterial defect that intermittently ruptures and clots off, forming a collection of blood products surrounded by the renal parenchyma or the renal capsule [5]. RAPs occur due to injury to the kidney following abdominal trauma or renal surgery [6, 7]. We present the case of an incidentally discovered renal artery aneurysm and review some of the literature surrounding the etiology, presentation, and management of both RAAs and RAPs.

## Case presentation

**Fig. 1:**
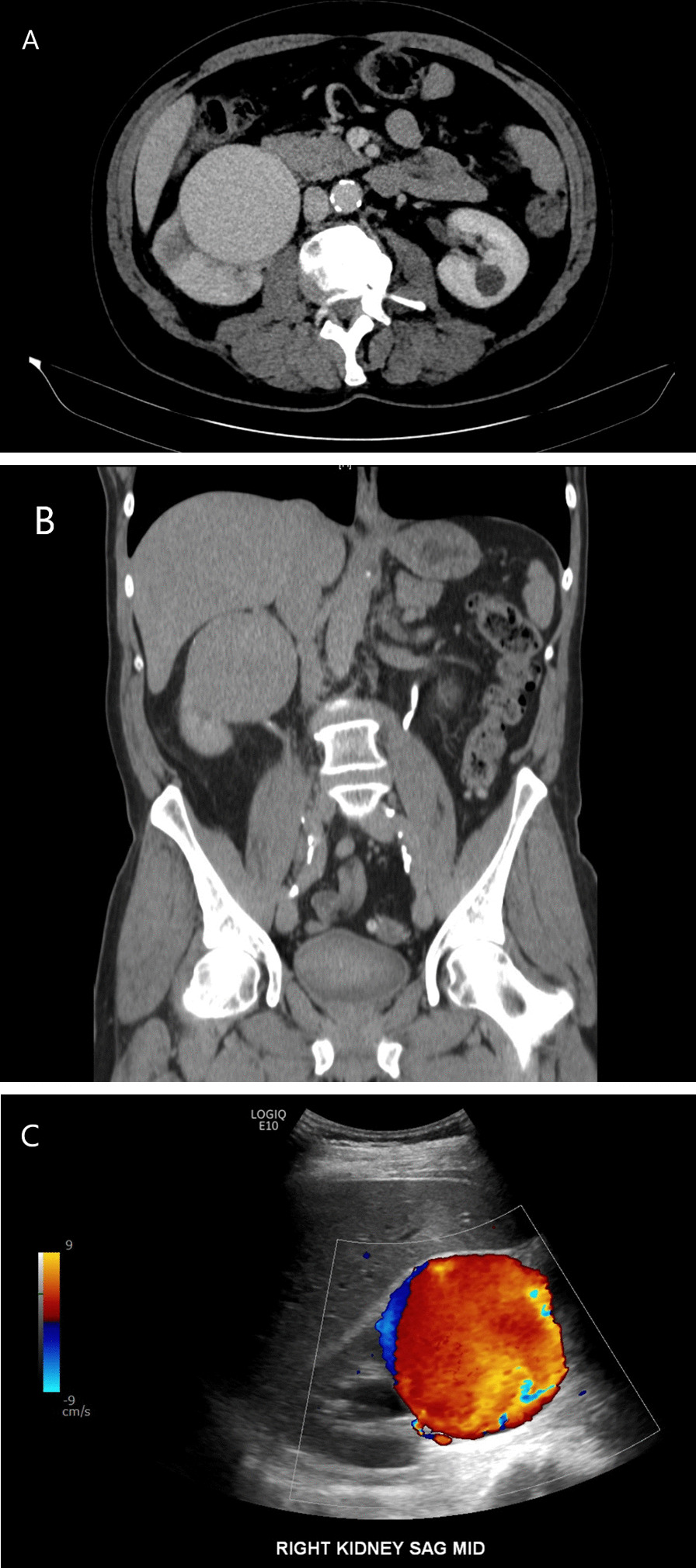
CT with intravenous contrast (**a**, **b**) demonstrated a 7.5 × 7.2 cm ovoid mass that was compressing on the upper pole and interpolar regions of the right kidney. The mass had similar density to blood. Doppler ultrasound (**c**) showed pulsatile flow within the right renal mass

A 62-year-old man with a 40-pack-year smoking history was referred to the urologic oncology clinic for workup of an incidentally discovered renal mass. After undergoing chest imaging for lung cancer screening, a right renal mass was found. Follow-up computed tomography (CT) imaging demonstrated a 7.5 × 7.4 × 7.5 cm well-circumscribed mass arising from the mid right kidney that enhanced homogenously with few calcifications (Fig. 1a, b). He did not have any recent surgeries or traumas. He was instructed to complete imaging work-up by obtaining a doppler ultrasound study. The ultrasound was completed on the same day and demonstrated a large vascular lesion with pulsatile flow concerning for a renal pseudoaneurysm (Fig. 1c). The patient was informed of the results and told to return promptly to the hospital for admission. After arriving, Interventional Radiology (IR) performed a renal angiogram which demonstrated a large aneurysm arising near the bifurcation of the right upper pole artery. IR attempted placement of a covered stent, but there was no good landing zone. A coil embolization was also considered, but it was ultimately deferred given the risk for infarction of a large portion of the right kidney.

Given the possible need for vascular reconstruction, the patient was transferred to the vascular surgery service at a neighboring hospital. The vascular team discussed options with the patient including open surgical reconstruction or endovascular intervention. Open surgery could involve auto-transplantation of the kidney after ex vivo reconstruction of the aneurysm. Another option is local repair of the aneurysm in-situ with aneurysm excision and reimplantation of the superior pole branch of the renal artery. Alternatively, endovascular approaches including placement of a covered stent or renal angioembolization could also be performed. Ultimately, the patient and the vascular team agreed to repeat an attempt for placement of a covered stent.

**Fig. 2:**
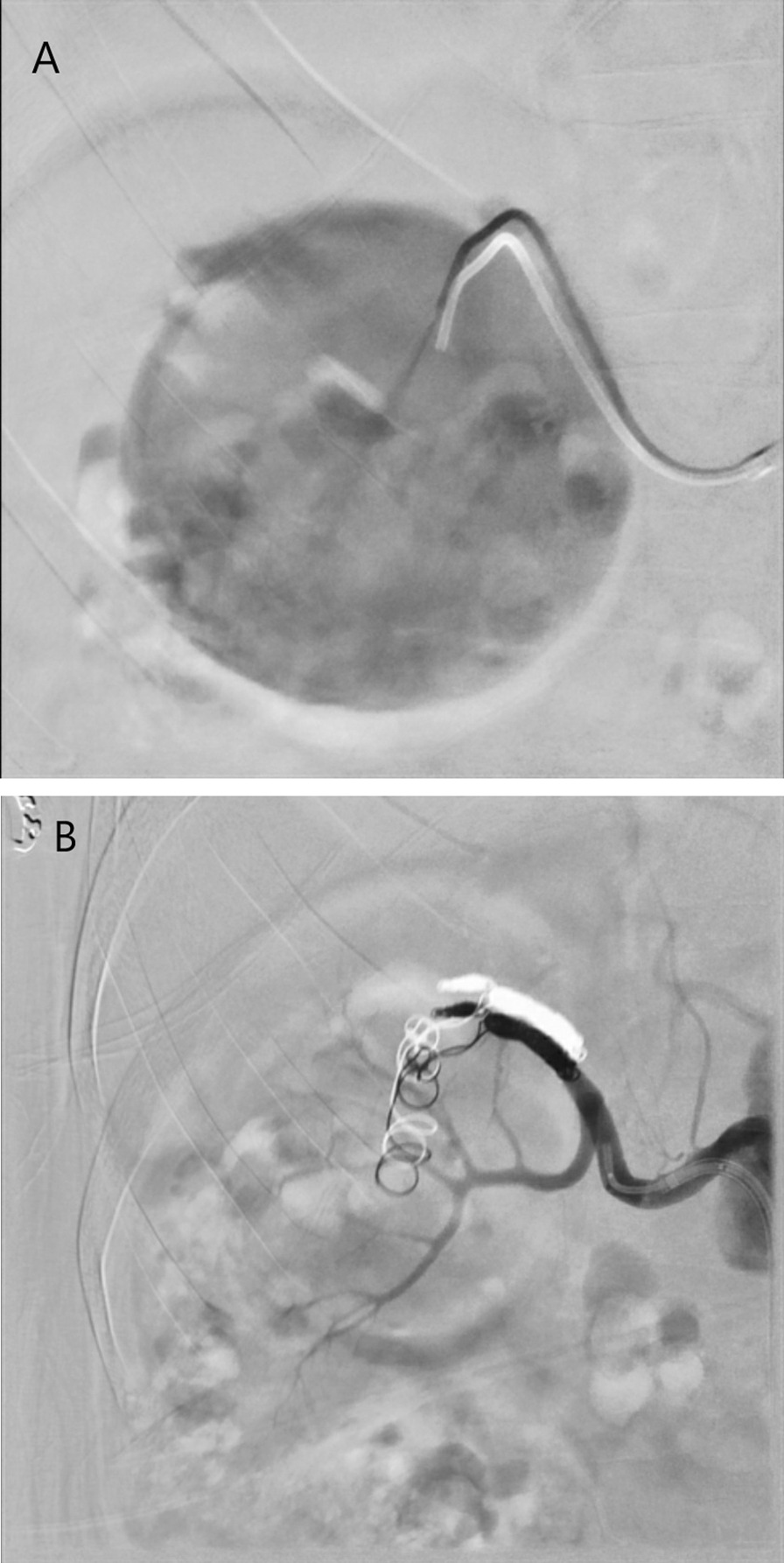
Right renal angiogram (**a**) revealed a 7 cm aneurysm. A superior pole branch of the right renal artery directly fed into the aneurysm. Selective coil embolization (**b**) excluded the inflow and outflow tracts for the aneurysm within the superior pole branch of the right renal artery. The inferior pole branch of the renal artery was preserved which allowed perfusion to a majority of the right kidney

Selective angiogram of the right renal artery confirmed a renal artery aneurysm originating from the superior pole branch (Fig. 2a). Vascular surgery was able to pass a microwire and catheter into the superior pole artery but was unable to advance a larger wire that would facilitate the placement of a covered stent. The decision was made to proceed with coil embolization of the outflow and inflow tract. Subsequent angiography demonstrated good exclusion of flow into the aneurysm with maintained flow into the inferior renal pole branch (Fig. 2b). The patient tolerated the procedure well and was discharged from the hospital on postoperative day 1.

**Fig. 3:**
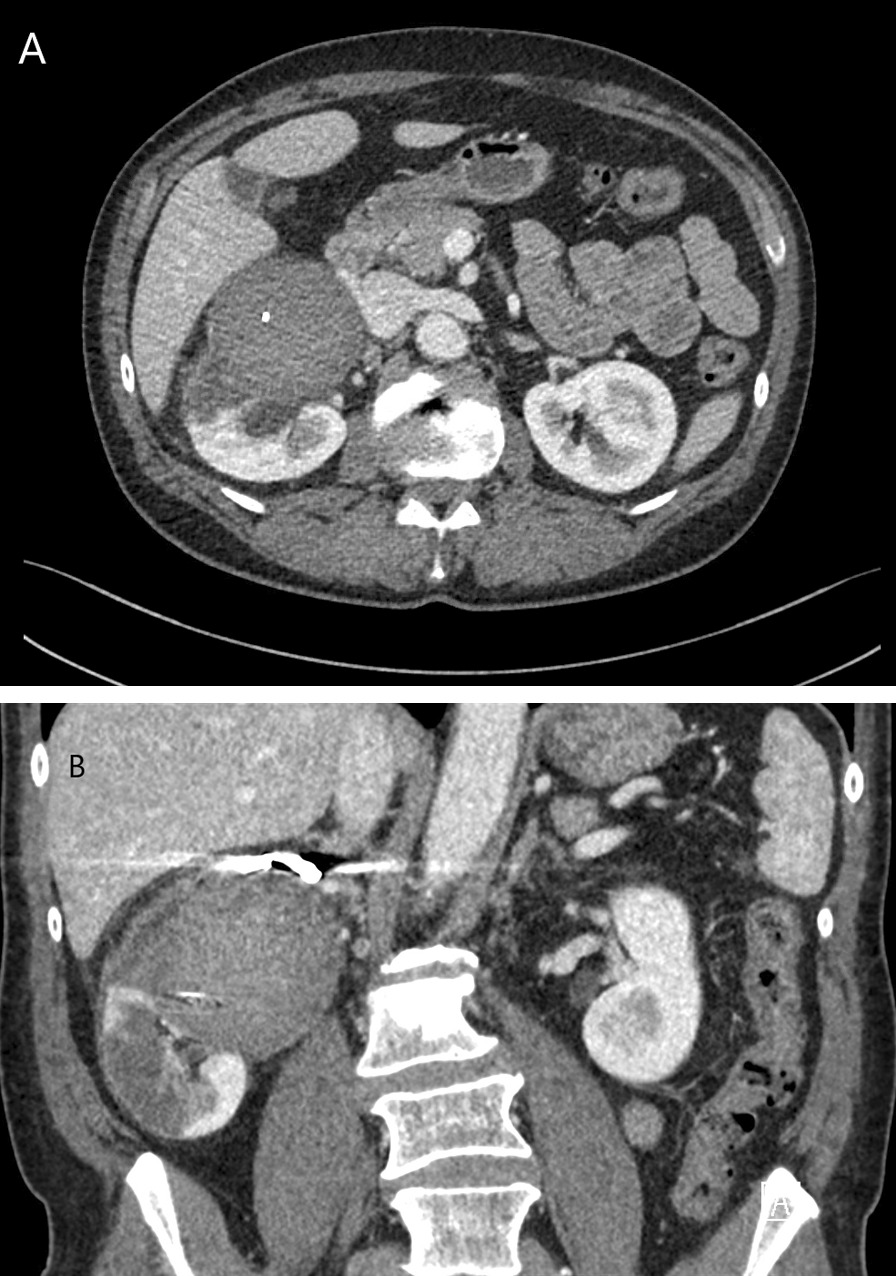
CTA (**a**, **b**) performed on postoperative day 14 demonstrated a 7.5 cm aneurysm with no contrast enhancement on arterial or delayed phases of imaging. There was hypoperfusion of the superior, anterior, and lateral portions of the right kidney indicating renal infarct

**Fig. 4:**
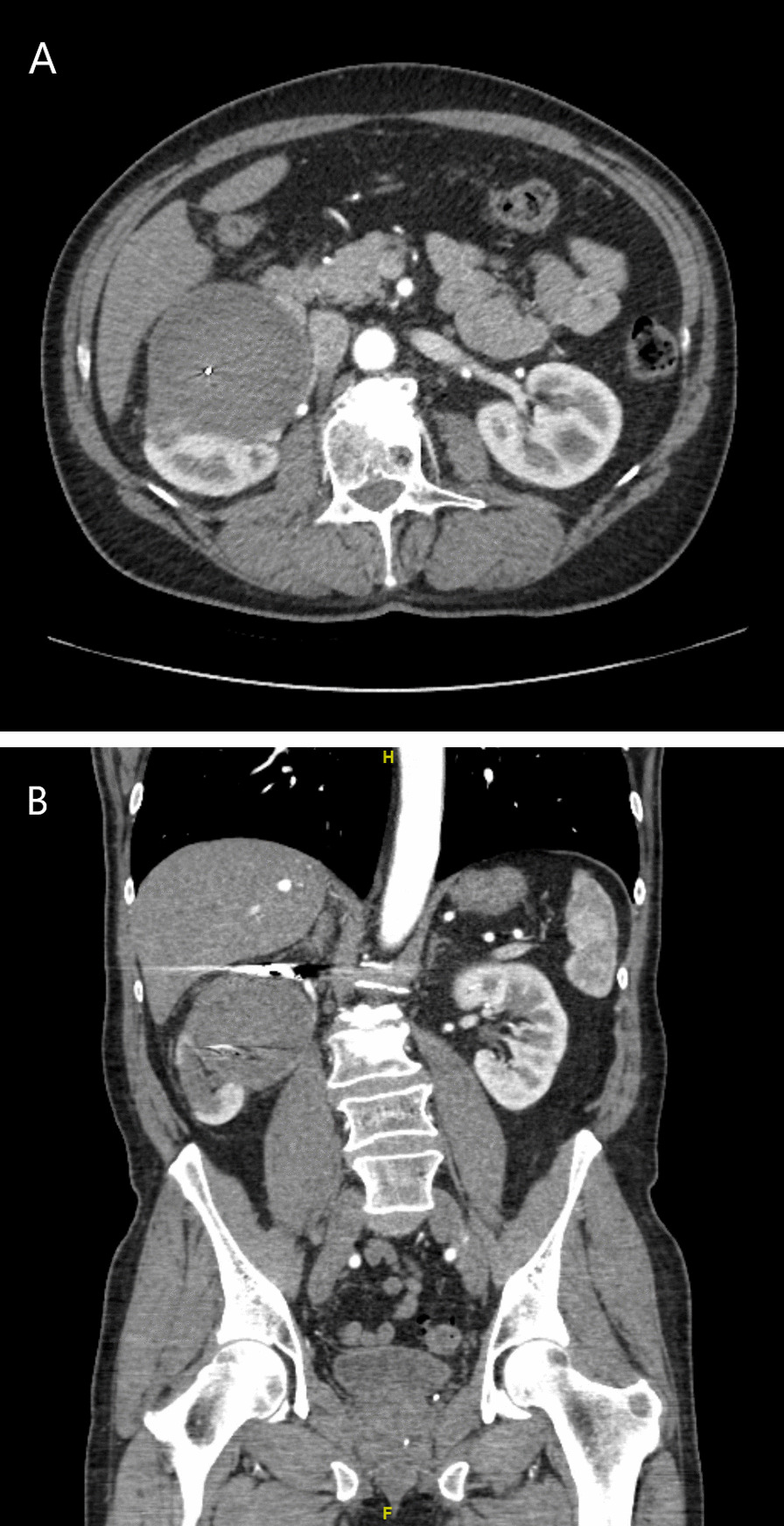
CTA (**a**, **b**) performed 3 months after angioembolization showed stable size of aneurysm sac. There was interval atrophy of right renal parenchyma due to renal infarct

The patient presented for outpatient follow-up on postoperative day 14 with new symptoms of nausea, abdominal pain, night sweats, and chills. He noted that his symptoms of nausea occurred specifically after eating fatty meals. Laboratory work showed no leukocytosis, anemia, or elevated creatinine, but there was a mild elevation in alanine aminotransferase. CT angiography (CTA) was performed and demonstrated metallic coiling in the right renal artery and a 7.5 cm aneurysm without contrast enhancement on arterial or delayed phases of imaging (Fig. 3a, b). Hypoperfusion of some areas of the kidney were also seen, consistent with infarction. At a 3-month follow-up visit, the patient reported feeling well and had resolution of prior symptoms. Repeat CTA showed stable size of the excluded renal aneurysm sac along with interval atrophy of the renal parenchyma consistent with prior renal infarct (Fig. 4a, b). Basic hematology and chemistry lab values were within normal limits. The patient is scheduled to return for additional follow-up 6 months after his procedure.

## Discussion and conclusion

Our case report demonstrates a patient who presented with an incidental finding of a RAA during workup for a renal mass. Although the renal lesion was initially diagnosed as a RAP by doppler ultrasonography, the subsequent angiography study revealed that the patient had a true aneurysm. RAAs are a rare phenomenon with an overall incidence of 0.09% within the general population [8]. They can be classified into three different forms: type 1 consists of saccular aneurysms arising from the main renal artery or a large segmental branch, type 2 includes fusiform aneurysms, and type 3 includes intralobar artery aneurysms [9]. Our patient presented with a saccular type aneurysm, which accounts for about 94% of all aneurysms [10].

RAAs typically present without symptoms, but as they grow in size, they can lead to pain, hematuria, and hypertension due to compression of the renal parenchyma [11]. Additionally, RAAs typically present after 60 years of age, are more commonly seen in females than males, are associated with hypertension, and are predominantly found on the right side when presenting as a unilateral lesion [12]. RAAs can be diagnosed on physical exam as a pulsatile mass in the region of the renal hilum or when heard as a bruit on auscultation [13]. However, these exam findings are rare and RAAs are more commonly found incidentally on imaging [1]. Our patient was initially diagnosed with a pseudoaneurysm given the presence of a “yin-yang sign” on doppler sonography, which is depicted by the swirling motion pattern of arterial blood flow in a cystic structure. This finding on doppler ultrasound is characteristic of a pseudoaneurysm, but it can also be present in a saccular aneurysm [14]. When evaluating for RAA, the most commonly used imaging modality is CTA, followed by non-contrast enhanced CT, then magnetic resonance angiography (MRA), then catheter angiography, and lastly ultrasound [1]. Current vascular surgery guidelines recommend the use of CTA as the primary imaging modality of choice to evaluate patients with a suspected RAA [15]. Additionally, non-contrast MRA is recommended for evaluating RAA in patients who have increased radiation exposure risks (children, pregnant women) or contraindications to receiving contrast material (renal insuffiency, contrast allergy) [15]. Contrast-enhanced MRI has been shown to reliably detect renal aneurysms. However, they do not have the ability to show mural calcifications, and they have poor visualization of the external arterial wall when compared to CTA [16]. The additional use of maximum intensity projection or volume rendering reconstruction of CTA images can enhance the anatomic detail of the renal vasculature and can help identify aneurysms located at smaller branch vessels [17, 18]. In our patient, additional imaging with CTA was deferred due to high clinical suspicion for RAP, and additional imaging would not have changed the patient’s plan to undergo renal angiography. Current guidelines also recommend catheter-based angiography for preoperative planning and for improving visualization of the distal arterial branches that may not be well seen on conventional cross-sectional imaging [15].

Although RAAs and RAPs are rare, it is nevertheless important to rule out its presence during the workup of a new renal mass. Our patient presented for oncologic workup of a renal mass found on CT. But if doppler ultrasonography had been performed with the CT scan, an aneurysm would have been detected sooner and the patient would not have had a delay in his care. A study by Inci et al. reported a patient who underwent laparoscopic partial nephrectomy. On interval CT scan at a three month follow-up visit, there was a hyper-vascular lesion noted at the prior resection site which was reported as a recurrence of renal cell carcinoma. The patient was asymptomatic at that time and subsequently underwent radical nephrectomy for presumed cancer recurrence. However, the pathology was negative for cancer and instead demonstrated a RAP [19]. Another study reported a similar case with an asymptomatic patient who presented with recurrent renal masses after laparoscopic partial nephrectomy. However, a renal Doppler ultrasound was performed which revealed two RAPs, and the patient subsequently underwent successful angioembolization of the two RAPs [20]. Through our case and these prior reports, we hope highlight the importance of using multiple imaging modalities in the workup of a new renal mass.

A RAP is also a rare clinical occurrence that forms after an inciting event that causes injury to the renal vasculature, such as abdominal trauma, renal biopsy, percutaneous renal surgery, partial nephrectomy, or renal transplant, [6, 7, 21–26]. While a true aneurysm is a localized dilation involving all layers of the arterial wall, a renal pseudoaneurysm is a hematoma that forms from an arterial defect and is contained by the surrounding renal parenchyma or capsule [5]. Patients with a RAP will typically present with signs and symptoms of gross hematuria, flank pain, and/or anemia, but they can also be asymptomatic [27]. Patients who developed RAP after partial nephrectomies typically presented with signs and symptoms of delayed bleeding through the urinary tract. Most patients have at least one of three signs or symptoms: gross hematuria, flank pain, or anemia. While 5% of patients will present with all three symptoms, around 87% of patients will present with gross hematuria [27]. Most patients who develop symptoms will present around two weeks after renal surgery [27, 28].

A RAP is a rare complication following percutaneous nephrolithotomy with rates reported to occur in 0.3–0.7% of cases [7, 23, 29, 30]. Despite having low reported rates, the true incidence of RAP after percutaneous procedures is unknown and may be higher if patients were asymptomatic and did not present with signs or symptoms that would lead to further diagnostic testing. RAPs also develop at low rates after partial nephrectomies. However, the risk for developing RAP after partial nephrectomies appear to be different for open and laparoscopic surgeries. Albani et al. reported 3 patients who developed RAPs out of 698 (0.43%) who had an open partial nephrectomy [24]. Conversely, Singh et al. showed that 6 patients developed RAPs out of 345 (1.7%) who had laparoscopic partial nephrectomy [21]. Similar rates were reported by Ghoneim et al. who noted that 7 out of 1,160 (0.6%) patients who underwent open partial nephrectomy and 8 out of 301 (2.7%) patients who underwent laparoscopic partial nephrectomy were diagnosed with RAPs [28]. A systematic review examined 30 published series and case reports that included 5,229 cases of open or laparoscopic partial nephrectomies and found that rates of RAP were significantly more likely to occur after laparoscopic procedures compared to open procedures (1.96% vs 1.00%, *p* ≤ 0.001) [27]. While overall rates for RAP after open or laparoscopic partial nephrectomies remain low, it is unclear why RAP is more likely occur after laparoscopic surgeries. Various factors involving surgical techniques used within laparoscopic surgery may be a contributing factor: larger needles can cause greater tissue trauma, parenchymal approximation may not be as tight, and pneumoperitoneum may mask small vascular injuries that would otherwise bleed [28]. Additionally, it has been shown that laparoscopic partial nephrectomies for tumors in the central portion of the kidney are more likely to develop RAP compared to surgeries for tumors in the periphery of the kidney [31].

Treatment for a RAA or RAP is typically sought when the aneurysm is greater than 3 cm in size, in a woman of childbearing age, or in a patient with medically refractory hypertension [15]. The main reason for treatment is to reduce the risk for aneurysm rupture which can lead to life-threatening hemorrhage. Earlier reports estimated a high risk of RAA rupture ranging from 14 to 30% [32, 33]. Prior guidelines recommended repair of visceral artery aneurysms greater than 2 cm [34]. However, more recent reports suggest RAAs have a much lower risk for rupture, even in aneurysms greater than 2 cm in size. Tham et al. followed 69 patients with RAA conservatively over a mean of 4 years and found that none of these patients developed RAA ruptures [35]. A multicenter center study by Klausner et al. showed that out of a cohort of 547 patients with untreated RAA, the rupture rate was 0.55%, and ruptures only occurred in patients with aneurysms greater than 3 cm in size [1]. Pregnancy has been associated with a higher rate of aneurysm rupture, possibly due to changes in vascular flow and hormonal shifts that result in weakening of the arterial wall elastic tissue [12]. However, the true incidence of RAA within the pregnant population is unknown [12]. The presence of a RAA with renovascular hypertension is also an indication for treatment. Studies have shown that reconstruction of a RAA can improve or cure co-existing hypertension, and the benefit of repair on hypertension is more likely to occur in those with renovascular hypertension than in those without arterial stenosis [2, 36, 37].

Options for treatment of a RAA include open surgical repair or endovascular approaches. Open surgery includes ex-vivo reconstruction with auto-transplantation of the kidney or in-situ repair of the aneurysm. These open surgical approaches offer good vascular patency rates ranging 93–100% and long-term survival rates averaging 90% at 10 years [38–40]. Additionally, when these surgeries are performed in the appropriate patient, they are well tolerated with minimal morbidity and complication rates [2, 41, 42].

Endovascular treatments for RAA include deployment of a covered stent or renal angioembolization. Small series have shown that the endovascular approach is well-tolerated with reported success rates of 83–100% and variable complication rates of 13–60% [43, 44]. When comparing the endovascular approach to the open surgical approach, retrospective studies have suggested that the endovascular approach is associated with shorter hospitalizations, similar or fewer complications, and similar success rates [45, 46]. Additionally, no studies have reported a significant difference in mortality, perioperative morbidity, freedom from reintervention, or decline in renal function when comparing open and endovascular approaches to repairing RAAs [1, 45, 47].

Endovascular stenting or embolization is also the preferred approach for a RAP. In rare cases in which angioembolization is unsuccessful, open surgery with nephrectomy is necessary [27]. Angioembolization of a RAP has a high success rate ranging 93–96% in select series [27, 28]. The procedure is well tolerated with a complication rate of around 5%; most common complications include incomplete embolization, coil migration, and groin hematoma [48]. The patient in our report presented on postoperative day 14 with symptoms of nausea, abdominal pain, night sweats, and chills. These symptoms are consistent post-infarction syndrome (PIS). Around 74% of patients after renal angioembolization will experience PIS, which include symptoms of flank pain, nausea, or vomiting [48]. The vast majority of these symptoms are mild and self-limited. Loss of renal function is an additional adverse effect of angioembolization due to the devascularization of renal parenchyma and/or the use of iodinated contrast which may be nephrotoxic. However, the use of coaxial catheters and microcoils for superselective embolization can minimize the loss of renal parenchyma. The loss of renal tissue can range from 0 to 10% at 6 months post-embolization, but overall renal function is typically preserved in patients after angioembolization [49]. However, those with solitary kidneys may have increased risk for deterioration of renal function after embolization [50].

Our case report documents a unique case of an asymptomatic patient who presented for evaluation for a renal mass but was incidentally found to have a large renal artery aneurysm. A RAA is a rare clinical finding that can be managed with surveillance, open surgical reconstruction, or endovascular approaches. Given the size of the RAA and its risk for rupture, our patient underwent successful treatment with renal angioembolization and had minimal adverse effects following treatment. Although our patient presented for oncologic workup given initial imaging findings, additional testing with doppler ultrasound elucidated the presence of a renal aneurysm which allowed the patient to be directed to the proper therapy. In addition to reviewing the etiology, presentation, and management of both RAAs and RAPs, we also hope to use our case to highlight the importance of using multiple imaging modalities for the workup of renal masses.

## Data Availability

Not applicable.

## Abbreviations

CT: Computed tomography
CTA: Computed tomography angiography
IR: Interventional radiology
MRA: Magnetic resonance angiography
PIS: Post-infarction syndrome
RAA: Renal artery aneurysm
RAP: Renal artery pseudoaneurysm
